# Epidemiology of suicidal feelings in an ageing Swedish population: from old to very old age in the Gothenburg H70 Birth Cohort Studies

**DOI:** 10.1017/S2045796019000143

**Published:** 2019-04-01

**Authors:** M. M. Fässberg, B. Vanaelst, M. Jonson, T. R. Sterner, F. Ahlner, H. Wetterberg, L. Rydén, S. Kern, R. Sigström, A. Zettergren, I. Skoog, M. Waern

**Affiliations:** Neuropsychiatric Epidemiology Unit, Department of Psychiatry and Neurochemistry, Sahlgrenska Academy, Centre for Ageing and Health (AgeCap) at the University of Gothenburg, Gothenburg, Sweden

**Keywords:** Aged, 80 and over, cohort studies, geriatric psychiatry, suicidal ideation

## Abstract

**Aims:**

The first aim of this study was to provide prevalence suicidal feelings over time (past week, past month, past year and lifetime) in a population-based sample of old to very old adults without dementia. Does prevalence change with rising age? The second aim was to examine the fluctuation of suicidal feelings over time. How does this coincide with depression status?

**Methods:**

Data were derived from the Gothenburg H70 Birth Cohort Studies (the H70 studies) which are multidisciplinary longitudinal studies on ageing. A representative sample of adults in Gothenburg, Sweden with birth years 1901–1944 were invited to take part in a longitudinal health study on ageing and participated at one or more occasions during 1986–2014. The sample consisted of 6668 observations originating from 3972 participants without dementia between the ages of 70 and 108, including 1604 participants with multiple examination times. Suicidal feelings were examined during a psychiatric interview using the Paykel questions (life not worth living, death wishes, thoughts of taking own life, seriously considered taking life, attempted suicide).

**Results:**

Prevalence figures for suicidal feelings of any severity were as follows: past week 4.8%, past month 6.7%, past year 11.2% and lifetime 25.2%. Prevalence rates increased with age in the total group and in women but not in men. Suicidal feelings were common in participants with concurrent major or minor depression, but over a third of the participants who reported suicidal feelings did not fulfil criteria for these diagnoses nor did they present elevated mean depressive symptom scores. The majority of participants consistently reported no experience of suicidal feelings over multiple examination times, but fluctuation was more common in women compared with men.

**Conclusion:**

Suicidal feelings in late-life are uncommon in individuals without depression indicating that such behaviour is not a widespread, normative phenomenon. However, such feelings may occur outside the context of depression.

## Introduction

With a growing share of older adults in today's society and a particular rapid increase in the number of very old persons, population ageing has become a repeatedly described social and economic challenge facing policy makers all over the world (Eurostat, 2015; United Nations Department of Economic and Social Affairs – Population Division, 2017).

In most regions of the world, suicide rates are highest in persons above 70 years of age (World Health Organization, 2014). For example, compared with a worldwide age-standardised suicide rate of 11.4/100 000 inhabitants (World Health Organization, 2014), the suicide death rate of Swedish men aged 75 and older was shown to be 28/100 000 in 2016 (Socialstyrelsen, 2016). A wealth of studies have investigated correlates of suicide and death ideation in older people, indicating that complex interactions between biological, psychological, social, environmental and cultural factors may contribute to the risk for late-life suicide (Fassberg *et al*., 2012; World Health Organization, 2014; Fassberg *et al*., 2016; Stolz *et al*., 2016; Rymo *et al*., 2017; Conejero *et al*., 2018), with depression being a solid correlator (Corna *et al*., 2010; Vasiliadis *et al*., 2012; Park *et al*., 2014; Holmstrand *et al*., 2015; Stolz *et al*., 2016). However, studies focusing suicidal behaviour in centenarians are lacking.

Prevalence figures and risk factors for late-life suicidal behaviour are often presented for entire cohorts with an age range over several decades. Suicide-related phenomena may vary with age within an ageing population. Not much is known about suicidal feelings in individuals who have lived an entire century. Similarly, studies of late-life suicidal ideation and behaviours are seldom sex-stratified, as highlighted in recent research reviews conducted by the International Research Group on Suicide in Older Adults (Lapierre *et al*., 2011; Fassberg *et al*., 2012; Fassberg *et al*., 2016). Large studies are needed in order to examine age and sex differences in an ageing population.

Suicidal feelings can range from occasional feelings of life weariness and thoughts about one's own death to more intense active wishes or plans to end one's life. People expressing such feelings may be reaching out for help or support. Studying the presence of suicidal feelings may therefore serve as an early stage marker for suicidal behaviour in suicidality research (Baca-Garcia *et al*., 2011). Even though the prevalence of death and suicide ideation has varied greatly over studies due to cross-national variations and methodological differences (e.g. definitions of ‘old age’ and ‘suicidal feelings’, timeframe and measure used), prevalence figures over 20% have been demonstrated for older adults in certain European countries (Fassberg *et al*., 2014; Stolz *et al*., 2016).

However, as illustrated in Table 1, very few studies have investigated suicidal feelings in a general population of old to very old adults. Prevalence of past month of suicidal feelings in studies of persons aged 70 and above range from 3 to 17% (Skoog *et al*., 1996; Scocco *et al*., 2001; Jonson *et al*., 2012; Fassberg *et al*., 2013). Many previous studies are of cross-sectional design, and only a handful included centenarians. Thinking about and wishing for death may be regarded as common when natural death is imminent. However, we know little about these phenomena in the very old. Do such feelings increase with age and do they commonly occur also in those without depression? The current study allows us to examine how the prevalence of suicidal behaviour changes from old to very old age.

**Table 1.** Studies presenting prevalence rates of suicidal feeling in old adults, using the Paykel questions as suicidal feelings measure

Community-based population studies	Examination year	Age	Number of participants	Period	Previously published prevalence
General population prevalence study by Paykel *et al*. – USA (Paykel *et al*., 1974)	1969	60+^a^	156	Past year	9.0%
Longitudinal Aging Study Amsterdam – the Netherlands (Rurup *et al*., 2011)^b^	2005–2006	58–98^a^	1794	Lifetime	15.3% (item 2)
Padua study in over-65-year-old population – Italy (Scocco *et al*., 2001)	1996–1997	65+ 65–74 75–84 85+	611	Past month Past year Lifetime Past month Past year Lifetime Past month Past year Lifetime Past month Past year Lifetime	17% 9.2% 6.5% 7% 10% 18% 5% 7% 14% 13% 19% 21%
WHO/SUPREMISS study – Australia (De Leo *et al*., 2005)^b^	2000–2002	65–74 75+	1237 761	Lifetime Lifetime	15.9% (item 1) 6.1% (item 4) 2.3% (item 5) 14.5% (item 1) 6.9% (item 4) 1.6% (item 5)
Gothenburg H70 Birth Cohort Studies – Sweden (Skoog *et al*., 1996; Jonson et al., 2012; Fassberg et al., 2013)	2000 1986 1998–2007	70 85 97	560 345 269	Past month Past month Past month	2.9% 15.9% 11.5%

a Not stratified for age.

b Adapted version of the Paykel questions.

### Aims of the study

A first aim of this study is therefore to provide epidemiological data on suicidal feelings in a large population of old to very old adults without dementia and to examine differences in the prevalence of such feelings during past week, past month, past year and lifetime with rising age, including the study of centenarians. Another understudied facet of late-life suicidal behaviour being addressed in this study is the fluctuation of experiencing suicidal feelings over time (past week, past week, past year and lifetime): Do suicidal feelings in old to very old adults fluctuate over multiple examination times? Is more stable affirmative reporting to be expected in case of concurrent depressive symptoms, or are suicidal feelings on the other hand a normal and constant finding over ageing?

## Methods

### Participants

**Fig. 1.**
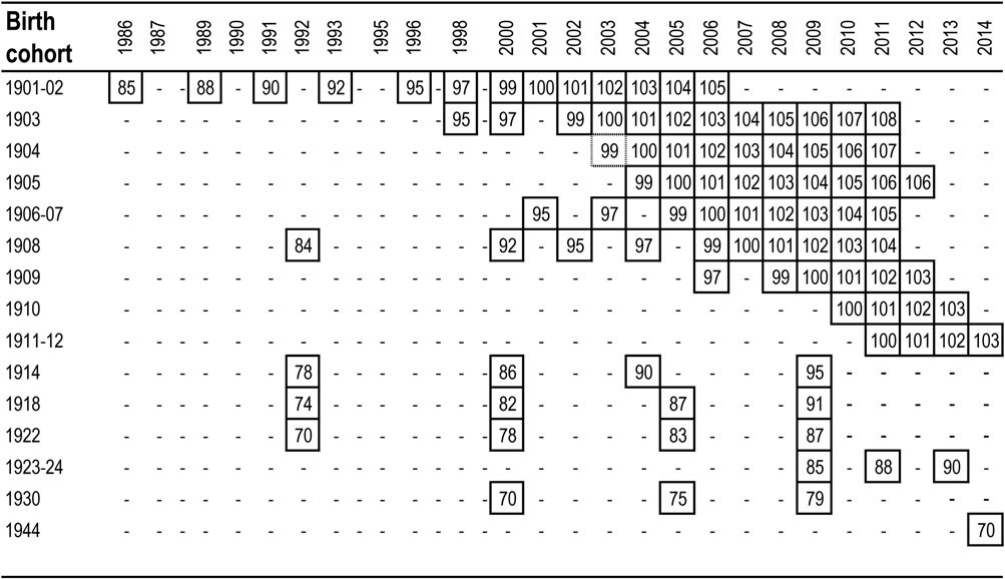
Overview of cohorts (age inside square) and examination years.

The Gothenburg H70 Birth Cohort Studies, which include the Prospective Population Study on Women and the Gothenburg 95+ study, are multidisciplinary, longitudinal health studies on ageing. All individuals born on specific dates, who were living in Gothenburg, Sweden, were invited to participate. The Swedish Population Register provided names and addresses. Persons living at home and in institutions were included in the study. Persons with insufficient knowledge of the Swedish language were excluded from the study. Participants were invited to take part in the study in connection with their birthday, thus interviews were spread over the calendar year. For more detailed information on sampling, participation and study methodology, see Bengtsson *et al*. (1997), Lissner *et al*. (2003), Rinder *et al*. (1975) and Skoog (2004). For study protocol on the cohort born in 1944, please see Rydberg Sterner *et al*. (2018). The studies were approved by the Ethics Committee for Medical Research at the University of Gothenburg and performed in accordance to the Declaration of Helsinki. Study participation followed after obtaining written informed consent. For persons with severe cognitive impairment, proxy consent was obtained by next-of-kin. Follow-up time varied depending on birth cohort, for information on year of birth, and age and year of examination, see Fig. 1.

The presented data originate from a sample of adults with birth years ranging from 1901 to 1944 and examination years from 1986 to 2014. This covers an age range from 70 to 108 years and allows analysing the following age groups: 70–79 year olds (mean age 73.2; median age 70), 80–89 year olds (mean age 85.4; median age 85), 90–99 year olds (mean age 94.7; median age 95) and 100–108 year olds (mean age 101.1; median age 101).

**Fig. 2.**
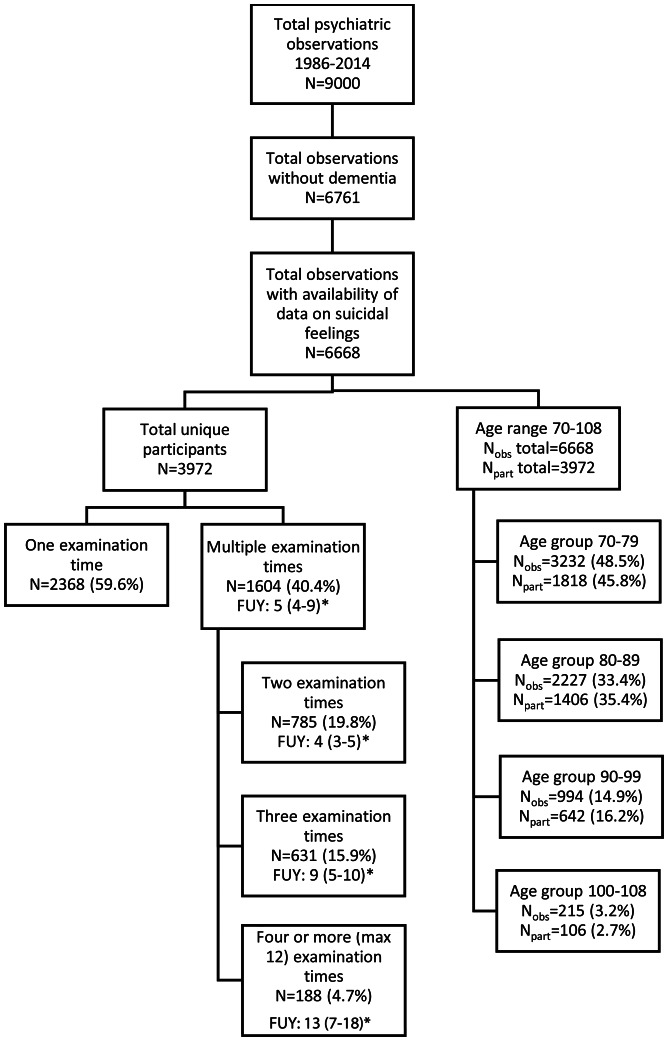
Flowchart of participants and observations. *FUY: follow-up years, median and 1^st^ and 3^th^ quartile of number of follow-up years. *N*_obs_: number of observations in specific age group, *N*_part_: number of unique participants with highest age at participation in that specific age group.

As illustrated in Fig. 2, the studied sample consisted of a total of 6668 observations originating from 3972 unique participants without dementia (i.e. inclusion was determined by availability of data on suicidal feelings and no dementia diagnosis). A participant could provide multiple observations on suicidal feelings given the longitudinal study design of the Gothenburg H70 Birth Cohort Studies. For instance, a participant who took part in the studies at age 70, 79, 85, 95 and 97 thus contributed as two observations in the age group 70–79, as one observation in the age group 80–89 and as two observations in the age group 90–99.

The majority of participants in the studied sample (59.6%) contributed one observation, whilst 19.8 and 15.9% of the participants provided two and three data points, respectively. For a minority of participants (4.7%), four or more data points are available (Fig. 2). In the followed-up participants, the number of follow-up years ranged from 1 to 19 with a median of 5 years.

### Procedures

Participants took part in a comprehensive battery of examinations including, e.g. psychiatric interviews, physical examinations and health interviews. The psychiatric interviews were conducted as semi-structured interviews by a psychiatrist, medical doctor or a mental health professional (the latter from year 2000 and onwards), either during a home visit or at the outpatient department. The semi-structured questions were nearly identical at each examination. Among those being selected for invitation over the examination years 1986–2014, participation rates varied between 57.9 and 73.4%. Dementia and depression were diagnosed using the Diagnostic and Statistical Manual of Mental Disorders DSM III R and DSM IV TR criteria as closely as possible (American Psychiatric Association, 1987, 2000), as described previously (Skoog, 2004; Guo *et al*., 2007; Skoog *et al*., 2015). Depression diagnosis was based on symptoms during the past month. The Montgomery Åsberg Depression Rating Scale (MADRS) (Montgomery *et al*., 1979), a subscale from the Comprehensive Psychopathological Rating Scale (Asberg *et al*., 1978), was used to rate depressive symptomatology. One-fourth (24.9%) of the observations were excluded from the analyses due to a dementia diagnosis at the time of examination (*N* = 2239, see Fig. 2). Death by suicide was established by the Cause of Death Register.

Suicidal feelings were assessed with the Paykel questions comprising four items on suicidal feelings of different intensity and one item on suicide attempts (Paykel *et al*., 1974). The following questions were asked: (1) ‘Have you ever felt that life was not worth living?’ (2) ‘Have you ever wished you were dead? For instance, that you could go to sleep and not wake up?’ (3) ‘Have you ever thought of taking your life, even if you would not really do it?’ (4) ‘Have you ever reached the point where you seriously considered taking your life, or perhaps made plans how you would go about doing it?’ (5) ‘Have you ever made an attempt to take your life?’ Responses to these questions were not mutually exclusive and participants with affirmative responses were asked to report the most recent occurrence of these feelings (with ‘past week’, ‘past month’, ‘past year’ and ‘a period longer than one year ago’ as possible alternatives). Inter-rater reliability was investigated among 113 individuals who had dual rating by psychiatric nurses and/or psychiatrists. The *κ* values for the individual Paykel items ranged from 0.96 (life weariness); 0.95 (death wishes); 0.83 (thoughts of taking own life); 0.74 (seriously considered taking life); to 0.49 (attempted suicide).

Recent time ranges were pooled into broader time periods (Paykel *et al*., 1974): *lifetime occurrence of suicidal feelings* was examined by grouping positive responses to any of the time periods (past week, past month, past year, longer than a year ago); *past year suicidal feelings* grouped the time periods past year, past month and past week; and *past month suicidal feelings* grouped the time periods past month and past week. In addition, a new variable was created representing suicidal feelings of any severity (an affirmative response to any of the five Paykel questions).

### Statistical analyses

Statistical analyses were performed using IBM SPSS, version 25. The prevalence of suicidal feelings was adjusted for age using the general Swedish population as standard population and using age distribution data on 7 age groups (70–74, 75–79, 80–84, 85–89, 90–94, 95–99, 100+) (Statistics Sweden, 2017). Pearson *χ*^2^ tests and Fisher's exact tests were used to analyse sex, age group and depression differences in the prevalence of suicidal feelings. A general linear model and generalised estimating equations were used to test for quadratic relation between age group and suicidal feelings in men. Suicidal feelings in the past month were the dependent variable and sex, age, age × men were independent variables. The same statistical model was used to test for interaction effects between sex and age group with suicidal feelings in the past month as the dependent variable and sex, male age and female age as independent variables. The fluctuation of experiencing suicidal feelings during the past week, past month, past year and/or lifetime was studied in participants with more than one examination time by creating three groups: (1) participants who reported no experience of suicidal feelings at any of the examined times (‘stable absence group’), (2) participants who affirmatively reported suicidal feelings at each of the examined times (‘stable presence group’) and (3) participants who reported experience of suicidal feelings at some but not all examined times (‘fluctuation group’). Kruskal–Wallis tests were used to examine mean age differences over the stable presence, the stable absence and the fluctuation group, with Mann–Whitney *U* tests being used as pairwise comparison test to investigate two-to-two differences. Results with a *p*-value <0.05 were considered significant, except for pairwise comparison testing where a Bonferroni correction was applied (*p*-value <0.016 considered significant for performing the two-to-two comparison tests in triple). Results are presented for the total group as well as for women and men separately.

## Results

### Sample characteristics

The mean age over all observations was 80.8 years (SD 9.29) (*N* = 6668, Fig. 2). Over two-thirds of the study observations (69.9%, *N* = 4663/6668) involved women; a similar proportion was noted for unique study participants (66.8%, *N* = 2652/3972). Major depression was present in 5.9% of the total observations (*N* = 393/6665), and in 8.6% of the unique participants at any of the examination times (*N* = 340/3972). The corresponding figures for minor depression were 11.6% (*N* = 771/6665) and 15.1% (*N* = 598/3972). Depression prevalence was greater in women compared with men (*p* < 0.001 for both major and minor depression). Figure 2 presents more detailed data on age-group distributions and the number of follow-up years for participants with multiple examination times. Three individuals died by suicide during the study period.

### Prevalence of suicidal feelings

The prevalence of past week, past month, past year and lifetime suicidal feelings (any severity) was 4.8, 6.7, 11.2 and 25.2%, as shown in Table 2. Prevalence figures were twice as high in the observations in women compared with men; differences were observed for any suicidal feeling, as well as specifically for feelings of life not worth living and death wishes over the past week, past month and past year (*p* < 0.001). When looking at the lifetime prevalence, women more frequently reported all of the questioned suicidal feelings, although this difference was not statistically significant for thoughts of taking one's own life. Prevalence figures for attempted suicide during the past week, past month and past year were low showing 0.03, 0.05 and 0.09% respectively. Lifetime prevalence of attempted suicide was 2%, and attempts were significantly more common in women than in men (*p* = 0.003).

**Table 2.** Prevalence of past week, past month, past year and lifetime suicidal feelings in adults aged 70–108 without dementia: unadjusted and age-adjusted percentages, and sex differences

	*N* total observations	Unadjusted %	Age-adjusted %^a^	*N* women (unadjusted %)	Women Age-adjusted %^a^	*N* men (unadjusted %)	Men Age-adjusted %^a^	*χ*^2^ (df)	*p*-value^‡^
Past week									
Any suicidal feeling	322/6668	4.83	3.70	269/4663 (5.77)	4.55	53/2005 (2.64)	2.22	29.8 (1)	<0.001
Life not worth living	273/6643	4.11	3.22	225/4647 (4.84)	3.90	48/1996 (2.40)	2.03	21.0 (1)	<0.001
Death wishes	233/6610	3.52	2.46	197/4636 (4.25)	3.07	36/1974 (1.82)	1.45	23.9 (1)	<0.001
Thoughts of taking own life	63/6600	0.95	0.56	48/4626 (1.04)	0.60	15/1974 (0.76)	0.50	1.1 (1)	0.288
Seriously considered taking own life	21/6426	0.33	0.16	15/4539 (0.33)	0.15	6/1887 (0.32)	0.16	0.0 (1)	0.936
Attempted suicide	2/6426	0.03	0.06	2/4539 (0.04)	0.00	0/1887 (0)	0.00	0.8 (1)	1.000^b^
Past month									
Any suicidal feeling	447/6668	6.70	5.54	372/4663 (7.98)	6.78	75/2005 (3.74)	3.30	40.2 (1)	<0.001
Life not worth living	378/6643	5.69	4.75	310/4647 (6.67)	5.71	68/1996 (3.41)	5.16	27.7 (1)	<0.001
Death wishes	312/6610	4.72	3.65	260/4636 (5.61)	4.49	52/1974 (2.63)	3.84	27.2 (1)	<0.001
Thoughts of taking own life	88/6600	1.33	0.98	65/4626 (1.41)	0.99	23/1974 (1.17)	1.90	0.6 (1)	0.436
Seriously considered taking own life	28/6426	0.44	0.28	19/4539 (0.42)	0.26	9/1887 (0.48)	0.80	0.1 (1)	0.746
Attempted suicide	3/6426	0.05	0.03	2/4539 (0.04)	0.01	1/1887 (0.05)	0.09	0.0 (1)	1.000^b^
Past year									
Any suicidal feeling	744/6668	11.16	10.02	607/4663 (13.02)	12.10	137/2005 (6.83)	5.97	54.0 (1)	<0.001
Life not worth living	621/6643	9.35	8.57	505/4647 (10.87)	10.30	116/1996 (5.81)	5.16	42.1 (1)	<0.001
Death wishes	508/6610	7.69	6.61	419/4636 (9.04)	8.06	89/1974 (4.51)	3.84	40.0 (1)	<0.001
Thoughts of taking own life	171/6600	2.59	2.15	128/4626 (2.77)	2.27	43/1974 (2.18)	1.90	1.9 (1)	0.168
Seriously considered taking own life	62/6426	0.96	0.80	43/4539 (0.95)	0.80	19/1887 (1.01)	0.80	0.0 (1)	0.824
Attempted suicide	6/6426	0.09	0.09	5/4539 (0.11)	0.10	1/1887 (0.05)	0.09	0.4 (1)	0.678^b^
Lifetime									
Any suicidal feeling	1677/6668	25.15	25.06	1297/4663 (27.81)	27.95	380/2005 (18.95)	18.57	58.4 (1)	<0.001
Life not worth living	1488/6643	22.40	22.62	1162/4647 (25.01)	25.47	326/1996 (16.33)	16.08	60.4 (1)	<0.001
Death wishes	1070/6610	16.19	15.70	851/4636 (18.36)	18.04	219/1974 (11.09)	10.65	53.8 (1)	<0.001
Thoughts of taking own life	594/6600	9.00	8.84	437/4626 (9.45)	9.13	157/1974 (7.95)	8.04	3.7 (1)	0.052
Seriously considered taking own life	311/6426	4.84	4.95	239/4539 (5.27)	5.34	72/1887 (3.82)	3.82	6.0 (1)	0.014
Attempted suicide	130/6426	2.02	2.22	107/4539 (2.36)	2.58	23/1887 (1.22)	1.24	8.7 (1)	0.003
**Population weights**			**1.00**						

a Age-adjusted prevalence reflects the prevalence of suicidal feelings that would have existed if the population under study had the same age distribution as the general Swedish 70+ population. ^‡^Based on *χ*^2^ test for sex differences (unadjusted).

b Results for Fisher's exact test.

Tables 3–5 show that past week, past month, past year and lifetime suicidal feelings were more prevalent among persons with depression at any examination compared with those without. However, suicidal feelings were also shown to occur in the absence of depression: in over a third of the observations with positive reporting of suicidal feelings, criteria for neither major nor minor depression were fulfilled (33.5% of the observations with past week suicidal feelings showed no depression diagnosis based on past month symptoms at any examination; the corresponding figure was 35.8% for past month suicidal feelings). Individuals not fulfilling criteria for depression may have elevated levels of depressive symptoms, this was however not the case in this study. The mean MADRS score (across examinations) was 0.96 in those with past week suicidal feelings but did not fulfil depression criteria at any examination.

**Table 3.** Prevalence of past week, past month, past year and lifetime suicidal feelings in adults aged 70–108 without dementia by depression at any examination

	Never depression		Minor depression		Major depression		Any depression		*χ*^2^ (df)	*p*-value^a^
	*N*	%	*N*	%	*N*	%	*N*	%		
Past week										
Any suicidal feeling	108/5501	1.96	93/771	12.06	121/393	30.79	214/1164	18.38	762.0 (2)	<0.001
Life not worth living	91/5483	1.66	77/769	10.01	105/388	27.06	182/1157	15.73	669.9 (2)	<0.001
Death wishes	74/5455	1.36	67/764	8.77	92/388	23.71	159/1152	13.80	601.8 (2)	<0.001
Thoughts of taking own life	18/5448	0.33	19/763	2.49	26/386	6.74	45/1149	3.92	177.8 (2)	<0.001
Seriously considered taking own life	7/5282	0.13	6/754	0.80	8/387	2.07	14/1141	1.23	29.5 (2)	<0.001^b^
Attempted suicide	2/5280	0.04	0/754	0	0/389	0	0/1143	0	–	–
Past month										
Any suicidal feeling	160/5501	2.91	127/771	16.47	160/393	40.71	287/1164	24.66	970.6 (2)	<0.001
Life not worth living	132/5483	2.41	105/769	13.65	141/388	36.34	246/1157	21.26	879.8 (2)	<0.001
Death wishes	97/5455	1.78	91/764	11.91	124/388	31.96	215/1152	18.66	832.5 (2)	<0.001
Thoughts of taking own life	22/5448	0.40	26/763	3.41	40/386	10.36	66/1149	5.74	299.8 (2)	<0.001
Seriously considered taking own life	8/5282	0.15	8/754	1.06	12/387	3.10	20/1141	1.75	46.5 (2)	<0.001^b^
Attempted suicide	2/5280	0.04	0/754	0	1/389	0.26	1/1143	0.09	3.4 (2)	0.207^b^
Past year										
Any suicidal feeling	313/5501	5.69	206/771	26.72	224/393	57.00	430/1164	36.94	1188.2 (2)	<0.001
Life not worth living	253/5483	4.61	170/769	22.11	197/388	50.77	367/1157	31.72	1079.5 (2)	<0.001
Death wishes	190/5455	3.48	144/764	18.85	173/388	44.59	317/1152	27.52	1016.1 (2)	<0.001
Thoughts of taking own life	56/5448	1.03	54/763	7.08	60/386	15.54	114/1149	9.92	372.1 (2)	<0.001
Seriously considered taking own life	21/5282	0.40	20/754	2.65	21/387	5.43	41/1141	3.59	120.8 (2)	<0.001
Attempted suicide	2/5280	0.04	2/754	0.27	2/389	0.51	4/1143	0.35	9.4 (2)	0.010^b^
Lifetime										
Any suicidal feeling	1059/5501	19.25	331/771	42.93	286/393	72.77	617/1164	53.01	704.7 (2)	<0.001
Life not worth living	953/5483	17.38	285/769	37.06	267/388	68.81	552/1157	47.71	666.2 (2)	<0.001
Death wishes	615/5455	11.27	226/764	29.58	228/388	58.76	454/1152	39.41	716.7 (2)	<0.001
Thoughts of taking own life	361/5448	6.63	124/763	16.25	108/386	27.98	232/1149	20.19	256.5 (2)	<0.001
Seriously considered taking own life	187/5282	3.54	62/754	8.22	62/387	16.02	124/1141	10.87	143.0 (2)	<0.001
Attempted suicide	66/5280	1.25	30/754	3.98	34/389	8.74	64/1143	5.60	118.9 (2)	<0.001

a Based on *χ*^2^ test for group differences in participants without depression, with minor depression and with major depression.

b Results for Fisher's exact test.

**Table 4.** Prevalence of past week, past month, past year and lifetime suicidal feelings in women aged 70–108 without dementia by depression at any examination

	Never depression		Minor depression		Major depression		Any depression		*χ*^2^ (df)	*p*-value^a^
	*N*	%	*N*	%	*N*	%	*N*	%		
Past week										
Any suicidal feeling	94/3760	2.5	78/576	13.5	97/324	29.9	175/900	19.4	485.8 (2)	<0.001
Life not worth living	78/3748	2.1	64/575	11.1	83/321	4.8	147/896	16.4	418.7 (2)	<0.001
Death wishes	66/3743	1.8	57/571	10.0	74/319	23.2	131/890	14.7	384.2 (2)	<0.001
Thoughts of taking own life	15/3738	0.4	15/568	2.6	18/317	5.7	33/885	3.7	95.3 (2)	<0.001
Seriously considered taking own life	6/3652	0.2	4/564	0.7	5/320	1.6	9/884	1.0	14.9 (2)	<0.001^b^
Attempted suicide	2/3650	0.1	0/565	0.0	0/321	0.0	0/886	0.0	0.7 (2)	1.000^b^
Past month										
Any suicidal feeling	136/3760	3.6	107/576	18.6	129/324	39.8	236/900	26.2	632.5 (2)	<0.001
Life not worth living	110/3748	2.9	87/575	15.1	113/321	35.2	200/896	22.3	569.4 (2)	<0.001
Death wishes	85/3743	2.3	77/571	13.5	98/319	30.7	175/890	19.7	525.3 (2)	<0.001
Thoughts of taking own life	18/3738	0.5	20/568	3.5	27/317	8.5	47/885	5.3	157.0 (2)	<0.001
Seriously considered taking own life	7/3652	0.2	5/564	0.9	7/320	2.2	12/884	1.4	21.5 (2)	<0.001^b^
Attempted suicide	2/3650	0.1	0/565	0.0	0/321	0.0	0/886	0.0	0.7 (2)	1.000^b^
Past year										
Any suicidal feeling	260/3760	6.9	165/576	28.6	181/324	55.9	346/900	38.4	773.8 (2)	<0.001
Life not worth living	211/3748	5.6	134/575	23.3	159/321	49.5	293/896	32.7	694.2 (2)	<0.001
Death wishes	162/3743	4.3	118/571	20.7	138/319	43.3	256/890	28.8	650.3 (2)	<0.001
Thoughts of taking own life	44/3738	1.2	41/568	7.2	42/317	13.2	83/885	9.4	207.8 (2)	<0.001
Seriously considered taking own life	17/3652	0.5	12/564	2.1	14/320	4.4	26/884	2.9	57.4 (2)	<0.001
Attempted suicide	2/3650	0.1	2/565	0.4	1/321	0.3	3/886	0.3	5.7 (2)	0.054^b^
Lifetime										
Any suicidal feeling	799/3760	21.3	260/576	45.1	237/324	73.1	497/900	55.2	498.4 (2)	<0.001
Life not worth living	718/3748	19.2	220/575	38.3	223/321	69.5	443/896	49.4	460.7 (2)	<0.001
Death wishes	482/3743	12.9	181/571	31.7	187/319	58.6	368/890	41.3	488.0 (2)	<0.001
Thoughts of taking own life	261/3738	7.0	93/568	16.4	82/317	25.9	175/885	19.8	158.5 (2)	<0.001
Seriously considered taking own life	147/3652	4.0	45/564	8.0	47/320	14.7	92/884	10.4	76.4 (2)	<0.001
Attempted suicide	55/3650	1.5	27/565	4.8	25/321	7.8	52/886	5.9	66.9 (2)	<0.001

a Based on *χ*^2^ test for group differences in participants without depression, with minor depression and with major depression.

b Results for Fisher's exact test.

**Table 5.** Prevalence of past week, past month, past year and lifetime suicidal feelings in men aged 70–108 without dementia by depression at any examination

	Never depression		Minor depression		Major depression		Any depression		*χ*^2^ (df)	*p*-value^a^
	*N*	%	*N*	%	*N*	%	*N*	%		
Past week										
Any suicidal feeling	14/1741	0.8	15/195	7.7	24/69	34.8	39/264	14.8	314.1 (2)	<0.001
Life not worth living	13/1735	0.7	13/194	6.7	22/67	32.8	35/261	13.4	299.8 (2)	<0.001
Death wishes	8/1712	0.5	10/193	5.2	18/69	26.1	28/262	10.7	256.6 (2)	<0.001
Thoughts of taking own life	3/1710	0.2	4/195	2.1	8/69	11.6	12/264	4.5	119.4 (2)	<0.001
Seriously considered taking own life	1/1630	0.1	2/190	1.1	3/67	4.5	5/257	1.9	43.1 (2)	<0.001
Attempted suicide	0/1630	0.0	0/189	0.0	0/68	0.0	0/257	0.0	–	–
Past month										
Any suicidal feeling	24/1741	1.4	20/195	10.3	31/69	44.9	51/264	19.3	375.0 (2)	<0.001
Life not worth living	22/1735	1.3	18/194	9.3	28/67	41.8	46/261	17.6	344.4 (2)	<0.001
Death wishes	12/1712	0.7	14/193	7.3	26/69	37.7	40/262	15.3	371.4 (2)	<0.001
Thoughts of taking own life	4/1710	0.2	6/195	3.1	13/69	18.8	19/264	7.2	206.2 (2)	<0.001
Seriously considered taking own life	1/1630	0.1	3/190	1.6	5/67	7.5	8/257	3.1	33.0 (2)	<0.001^b^
Attempted suicide	0/1630	0.0	0/189	0.0	1/68	1.5	1/257	0.4	8.7 (2)	0.036^b^
Past year										
Any suicidal feeling	57/1741	3.0	41/195	21.0	49/69	62.3	84/264	31.8	434.6 (2)	<0.001
Life not worth living	42/1735	2.4	36/194	18.6	38/67	56.7	74/261	28.4	411.1 (2)	<0.001
Death wishes	28/1712	1.6	26/193	13.5	35/69	50.7	61/262	23.3	221.0 (2)	<0.001
Thoughts of taking own life	12/1710	0.7	13/195	6.7	18/69	26.1	31/264	11.7	88.9 (2)	<0.001
Seriously considered taking own life	4/1630	0.2	8/190	4.2	6/67	10.4	15/257	5.8	8.7 (2)	<0.001^b^
Attempted suicide	0/160	0.0	0/189	0.0	1/68	1.5	1/257	0.4	–	–
Lifetime										
Any suicidal feeling	260/1741	14.9	71/195	36.4	49/69	71.0	120/264	45.5	178.7 (2)	<0.001
Life not worth living	217/1735	12.5	65/194	33.5	44/67	65.7	109/261	41.8	179.8 (2)	<0.001
Death wishes	133/17121	7.8	45/193	23.3	41/69	59.4	86/262	32.8	211.7 (2)	<0.001
Thoughts of taking own life	100/1710	5.8	31/195	15.9	26/69	37.7	57/264	21.6	110.4 (2)	<0.001
Seriously considered taking own life	40/1630	2.5	17/190	8.9	15/67	22.4	32/257	12.5	84.8 (2)	<0.001
Attempted suicide	11/1630	0.7	3/189	1.6	9/68	13.2	12/257	4.7	34.6 (2)	<0.001

a Based on *χ*^2^ test for group differences in participants without depression, with minor depression and with major depression.

b Results for Fisher's exact test.

Suicide attempts were relatively uncommon in all groups with a lifetime prevalence of 1.25% for those with no depression at any examination and 5.60% for those with any depression at any examination (*p* < 0.001).

**Fig. 3.**
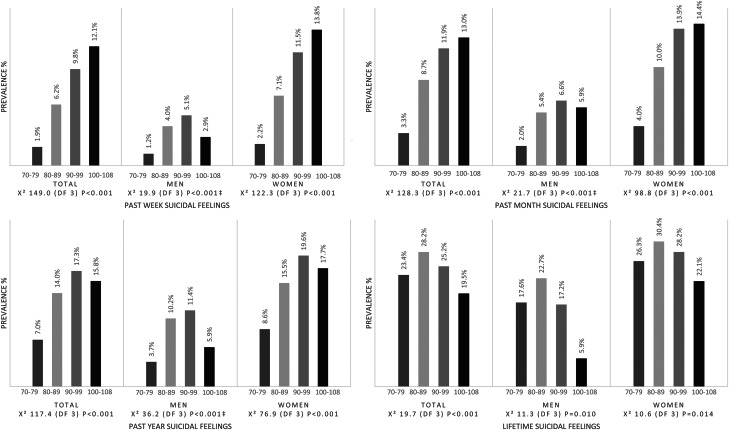
Prevalence of past week, past month, past year and lifetime suicidal feelings in adults aged 70–108 without dementia: age group differences. Age group 70–79: total *N* observations = 3232, *N* observations in men = 1068, *N* observations in women = 2164; age group 80–89: total *N* observations = 2227, *N* observations in men = 630, *N* observations in women = 1597; age group 90–99: total *N* observations = 994, *N* observations in men = 273, *N* observations in women = 721; age group 100–108: total *N* observations = 215, *N* observations in men = 34, *N* observations in women = 181; *p*-values for *χ*^2^ statistics for age group differences. ^‡^Results for Fisher's exact test.

As illustrated in Fig. 3, the prevalence of past week and past month suicidal feelings (any severity) increased with increasing age in the total group and for women. The prevalence among men between the ages of 100 and 108 seems to decrease. However, the risk for suicidal feelings among men was similar across the four age groups, as measured by the quadratic relation (OR 1.00, Wald *χ*^2^ = 1.83, df = 1, 95% CI 0.99–1.00, *p* = 0.176). Further, an interaction term between sex and age group on suicidal feelings was found non-significant (OR 0.77, Wald *χ*^2^ = 0.92, df = 1, 95% CI 0.15–3.92, *p* = 0.154), therefore a common age effect is reported for both sexes (OR 4.00, Wald *χ*^2^ = 20.02, df = 1, 95% CI 2.18–7.35, *p* < 0.001). Figure 3 shows a decline of past year and lifetime suicidal feelings within both sexes. Prevalence of lifetime suicidal feelings was highest in the age group 80–89, thus not demonstrating an increasing prevalence with rising age.

### Suicidal feelings over time

Among participants with multiple examinations (*N* = 1604), the vast majority consistently reported no experience of suicidal feelings in the past week, the past month or the past year. Suicidal feelings were absent at all examinations for over half of those with repeated examinations and stable affirmative reporting of suicidal feelings over the examinations years was shown to be rare (Tables 6–8). Fluctuation in the experience of past week, past month, past year and lifetime suicidal feelings was observed in 8.7, 12.2, 18.0 and 28.0% of the participating older adults (‘fluctuation group’), with a higher percentage of fluctuation in women (*p* < 0.001). More closely, transitions from reported affirmative suicidal feelings to reported negative suicidal feelings were observed in 45.2, 44.4, 40.7 and 39.1% of participants reporting past week, past month, past year and lifetime suicidal feelings. There was no difference between the sexes in regards to the prevalence of inconsistently reporting lifetime suicidal feelings (39.5% men *v*. 39.0% women, *χ*^2^ = .014, df = 1, *p* = 0.905). However, those with inconsistent reports of lifetime suicidal feelings were more often found in those without depression at all examinations compared with those with consistent reports of suicidal feelings (46.4 *v*. 29.4, *χ*^2^ = 19.2, df = 1, *p* < 0.001).

**Table 6.** Stability and fluctuation of experiencing suicidal feelings (any severity) in participants with multiple follow-up examinations: mean age, sex and depression status (at any examination) differences

Participants with multiple follow-up examinations	*N* of unique participants (median number of examination times)	Any suicidal feeling past week					Any suicidal feeling past month					Any suicidal feeling past year					Any lifetime suicidal feelings				
		% Absence	% Presence	% Fluctuation	*χ*^2^ (df)	*p*-value^a^	% Absence	% Presence	% Fluctuation	*χ*^2^ (df)	*p*-value^a^	% Absence	% Presence	% Fluctuation	*χ*^2^ (df)	*p*-value^a^	% Absence	% Presence	% Fluctuation	*χ*^2^ (df)	*p*-value^a^
Total	1604 (3)	90.1	1.1	8.7		NA	86.3	1.4	12.2		NA	78.9	3.0	18.1		NA	59.7	12.3	28.0		NA
Men	451 (2)	95.1	0.7	4.2	17.5 (2)	<0.001	92.7	1.1	6.2	21.9 (2)	<0.001	87.4	2.4	0.2	28.2 (2)	<0.001	71.2	9.8	19.1	35.1 (2)	<0.001
Women	1153 (3)	88.2	1.3	10.5			83.9	1.6	14.6			75.5	3.2	21.2			55.2	13.4	31.5		
Depression	524 (3)	76.0	3.1	21.0	177.3 (2)	<0.001	68.1	4.0	27.9	221.7 (2)	<0.001	53.8	8.0	38.2	299.5 (2)	<0.001	35.3	22.1	42.6	196.8 (2)	<0.001
No depression ^b^	1080 (2)	97.0	0.2	2.8			95.2	0.2	4.6			91.0	0.6	8.4			71.5	7.6	20.9		
Mean age (SD) Absence–presence^c^ Presence–fluctuation^c^ Absence–fluctuation^c^	NA	81.8 (7.47)	87.6 (9.32)	86.8 (7.89)	62.8 (2) 8655.0 1251.5 62333.5	<0.001^d^ 0.013^c^ 0.963^c^ <0.001	81.7 (7.46)	86.4 (9.51)	85.5 (8.08)	54.1 (2) 11951.5 2235.5 93519.0	<0.001^d^ 0.037^c^ 0.948^c^ <0.001^c^	81.7 (7.49)	85.1 (8.88)	84.2 (7.91)	36.2 (2) 24466.0 6959.0 144946.0	<0.001^d^ 0.020^c^ 0.968^c^ <0.001^c^	82.0 (7.67)	81.4 (7.35)	83.21 (7.78)	23.2 (2) 89083.0 35681.0 185 559.0	0.004^d^ 0.178^c^ <0.001^c^ <0.001^c^

a *χ*^2^ statistics.

b Depression diagnosis at one or more examination times *v.* never diagnosed with depression over all examination times.

c Two-to-two analyses by multiple range Mann–Whitney *U* tests and Bonferroni corrected *p*-value = 0.016 for significance.

d Kruskal–Wallis test.

**Table 7.** Stability and fluctuation of experiencing suicidal feelings (any severity) in women with multiple follow-up examinations: mean age, sex and depression status (at any examination) differences

Participants with multiple follow-up examinations	*N* of unique participants (median number of examination times)	Any suicidal feeling past week					Any suicidal feeling past month					Any suicidal feeling past year					Any lifetime suicidal feelings				
		% Absence	% Presence	% Fluctuation	*χ*^2^ (df)	*p*-value^a^	% Absence	% Presence	% Fluctuation	*χ*^2^ (df)	*p*-value^a^	% Absence	% Presence	% Fluctuation	*χ*^2^ (df)	*p*-value^a^	% Absence	% Presence	% Fluctuation	*χ*^2^ (df)	*p*-value^a^
Depression	414 (3)	74.2	3.1	22.7	123.0 (2)	<0.001	68.2	3.9	30.0	150.9 (2)	<0.001	51.1	8.0	40.3	205.0 (2)	<0.001	32.9	22.2	44.9	133.3 (2)	<0.001
No depression^b^	739 (3)	96.1	0.3	3.7			93.8	0.3	6.0			88.9	0.5	10.6			67.7	8.4	24.0		
Mean age (SD) Absence–presence^c^ Presence–fluctuation^c^ Absence–fluctuation^c^	NA	85.5 (6.84)	90.6 (8.87)	90.2 (7.08)	48.8 (2) 5112.0 879.0 38 744.0	<0.001^d^ 0.027^c^ 0.843^c^ <0.001^c^	85.5 (6.82)	89.4 (8.76)	89.3 (7.32)	39.2 (2) 6478.5 1494.0 57 587.5	<0.001^d^ 0.061^c^ 0.934^c^ <0.001^c^	85.5 (6.83)	88.3 (8.15)	88.0 (7.30)	24.7 (2) 12 930.0 4458.5 85 878.0	<0.001^d^ 0.040^c^ 0.872^c^ <0.001^c^	85.7 (6.98)	85.0 (6.89)	87.2 (7.14)	16.0 (2) 45 711.5 22 668.5 100 886.5	<0.001^d^ 0.195^c^ <0.001^c^ <0.001^c^

a *χ*^2^ statistics.

b Depression diagnosis at one or more examination times *v.* never diagnosed with depression over all examination times.

c Two-to-two analyses by multiple range Mann–Whitney *U* tests and Bonferroni corrected *p*-value = 0.016 for significance.

d Kruskal–Wallis test.

**Table 8.** Stability and fluctuation of experiencing suicidal feelings (any severity) in men with multiple follow-up examinations: mean age, sex and depression status (at any examination) differences

Participants with multiple follow-up examinations	*N* of unique participants (median number of examination times)	Any suicidal feeling past week					Any suicidal feeling past month					Any suicidal feeling past year					Any lifetime suicidal feelings				
		% Absence	% Presence	% Fluctuation	*χ*^2^ (df)	*p*-value^a^	% Absence	% Presence	% Fluctuation	*χ*^2^ (df)	*p*-value^a^	% Absence	% Presence	% Fluctuation	*χ*^2^ (df)	*p*-value^a^	% Absence	% Presence	% Fluctuation	*χ*^2^ (df)	*p*-value^a^
Depression	110 (2)	82.7	2.7	14.5	48.5 (2)	<0.001	75.5	4.5	20.0	64.7 (2)	<0.001	61.8	8.2	30.0	86.4 (2)	<0.001	44.5	21.8	33.6	52.3 (2)	<0.001
No depression^b^	341 (2)	99.1	0.0	0.3			98.2	0.0	1.8			95.6	0.6	3.8			79.8	5.9	14.4		
Mean age (SD) Absence–presence^c^ Presence–fluctuation^c^ Absence–fluctuation^c^	NA	84.5 (7.04)	87.3 (6.43)	89.8 (7.58)	8.6 (2) 442.0 26.5 2591.0	0.013^d^ 0.328^c^ 0.857^c^ 0.005^c^	84.4 (7.02)	87.0 (9.33)	89.1 (7.24)	9.8 (2) 855.5 64.5 3897.5	0.007^d^ 0.466^c^ 0.789^c^ 0.002^c^	84.4 (7.04)	86.4 (7.74)	87.4 (7.31)	6.0 (2) 1845.5 242.5 7231.5	0.049^d^ 0.380^c^ 0.829^c^ 0.019^c^	84.5 (7.14)	83.39 (6.30)	86.0 (7.40)	4.5 (2) 6315.5 1483.0 12 375.0	0.104^d^ 0.235^c^ 0.037^c^ 0.125^c^

a *χ*^2^ statistics.

b Depression diagnosis at one or more examination times *v.* never diagnosed with depression over all examination times.

c Two-to-two analyses by multiple range Mann–Whitney *U* tests and Bonferroni corrected *p*-value = 0.016 for significance.

d Kruskal–Wallis test.

Participants without a concurrent diagnosis of minor or major depression at any of the examination times most frequently reported a stable absence of suicidal feelings, whilst participants with any depression diagnosis had the highest percentage of consistent affirmative reporting or fluctuating experience of suicidal feelings in the past.

Significant age differences were observed between the reporting groups: participants with consistent absence of suicidal feelings over the examination years were significantly younger than the participants in the ‘stable presence’ or ‘fluctuation’ group. This finding was not observed in examining the transitions regarding lifetime suicidal feelings.

## Discussion

In this prospective study, we examined suicidal feelings in a large sample of older adults including centenarians. We presented age group-specific prevalence rates of suicidal feelings over four decades of ageing, demonstrating an increasing prevalence of suicidal feelings with increasing age. In addition, suicidal feelings were assessed over multiple examination years with a median duration of 5 years of follow-up. The vast majority of participants consistently reported no experience of suicidal feelings over multiple examination times, although fluctuation in experiencing suicidal feelings was an important finding.

### Prevalence comparisons

Due to a scarcity in previous studies using the Paykel questions in old populations, data evaluation is limited to the studies previously cited in Table 1. The observed prevalence percentages of lifetime suicidal feelings were higher in this study compared with the study by Scocco *et al*. (2001). However, prevalence of past month suicidal feelings were similar to the Italian study. Disparities may be due to methodological reasons, as our study includes both persons living at home as well as in institutions, whilst the study by Scocco *et al*. (2001) does not include the latter group. Prevalence of past year attempted suicide was lower in this study, compared with Paykel *et al*. (1974). Our group previously described prevalence of suicidal feelings on specific age bands in the Gothenburg H70 Birth Cohort Studies (Skoog *et al*., 1996; Jonson *et al*., 2012; Fassberg *et al*., 2013). If these percentages are fitted within the observed prevalence per decade of age (i.e. within the age groups 70–79, 80–89, 90–99, 100–108), they coincide very well, with one exception: the formerly published prevalence for past month suicidal feelings in a sample of 85 year olds was considerably higher (15.9%), most likely due to the more limited sample size (*N* = 345) (Skoog *et al*., 1996).

Apart from methodological heterogeneity which may cause diverging prevalence data, also cross-national variations account for variance in prevalence percentages: the prevalence of past month death wishes ranged from 6.9 to 21.1% between 12 European countries (Stolz *et al*., 2016) and from 3 to 27% in another study including 11 European centres (Fassberg *et al*., 2014). This implies caution in generalisation of the presented prevalence figures to other populations and/or other countries.

The presented results are in line with longitudinal data from the SHARE study, which indicated female sex, older age and depression to be important predictors in the development of passive suicidal ideation (Stolz *et al*., 2016). The majority of studies in the field indicate a higher prevalence of suicidal feelings in women (Skoog *et al*., 1996; Scocco *et al*., 2001; De Leo *et al*., 2005; Vasiliadis *et al*., 2012; Fassberg *et al*., 2013; Ciulla *et al*., 2014; Stolz *et al*., 2016). Clinical practice may support this sex difference in the observation that women more easily and openly communicate about feelings and sensitive topics (Luppa *et al*., 2012), such as, for example, suicide attempts. Also, a more frequent occurrence of psychiatric comorbidities in women may account for a higher prevalence of suicidal feelings (Skoog, 2011).

An increase in suicidal feelings was detected with increasing age for past week and past month suicidal feelings for the total group, in line with findings from previous studies in older populations (Barnow and Linden, 2000; Saias *et al*., 2012; Li *et al*., 2016; Stolz *et al*., 2016). Suffering from chronic pain, disability, social isolation, health deterioration and other challenges related to later life may explain this age increase in the prevalence of suicidal feelings. However, we did not observe an age-specific increase in men in the stratified analyses. Nor did we find increasing prevalence with age regarding lifetime suicidal feelings, which could potentially be explained by a survival effect and/or recall bias in the lifetime reporting variable.

Whilst both major and minor depression were strongly related to suicidal feelings, we could show that a third of those acknowledging past week suicidal feelings fulfilled criteria for neither of these diagnoses. A similar proportion was seen for past month suicidal feelings. These outcomes confirm previous findings from our research group (Fassberg *et al*., 2013) and highlight that suicidal feelings may occur outside the context of depression in very old adults, although this is uncommon.

### Suicidal feelings over time

This study prospectively examined the fluctuation of experiencing suicidal feeling over time and hereby contributes fundamentally to the field of late- and very late-life suicide. Inconsistent affirmative reporting of lifetime suicidal feelings was observed in over a quarter of the participants with multiple examination times, indicating that the experience of suicidal feelings fluctuates over time and with changing contexts (Witte *et al*., 2006; Stolz *et al*., 2016; Hallensleben *et al*., 2018).

One could assume that affirmative reporting of lifetime suicidal feelings would inevitably be repeated as an affirmative report at any future examination. However, a lack in acknowledging affirmative lifetime suicidal feelings at a next examination was observed in 39.1% of our study participants. This group mostly consisted of participants who had not fulfilled criteria for depression at any examination. Goldney *et al.* proposed a fail to recall, a conscious denial or an unconscious suppression of previous painful memories as possible clarifications for this phenomenon, and suggested that forgetting painful events such as suicidal ideation could be an adaptive defence mechanism, given their observations of a better mental health in study participants who disremembered suicidal feelings after a period of 4 years (Goldney *et al*., 2009).

Even though the overall prevalence of suicidal feelings ranged from 4.8% (past week suicidal feelings) to 25.2% (lifetime suicidal feelings), consistent affirmative reporting of suicidal feelings over time was uncommon and the prevalence of suicidal feelings was low in the absence of depression. We therefore suggest that suicidal feelings in old age are not a widespread phenomenon that reflects normative adjustments to the process of ageing. Van Orden *et al.* describe suicidal feelings in older adults as signals of life dissatisfaction and point to the importance of not withholding treatment for depression or suicide risk in older adults. The latter research group furthermore recommended to focus on the potential prognostic and clinical differences between having thoughts that life is not worth living and experiencing a desire for death: older adults expressing a desire for death may clinically be experiencing significant distress requiring appropriate treatment, whilst the experience that life is not worth living may basically represent less malign feelings of life dissatisfaction; both measures thus not being equivalent in their relationship to risk for suicide (Van Orden *et al*., 2015; Van Orden and Conwell, 2016).

### Strengths and limitations

This unique study setting allowed us to explore the prevalence of late-life suicidal feelings, with observations covering a broad age span in late- to very late-life, with prospective follow-up examinations and with assessments of suicidal feelings being performed by trained personnel during a psychiatric interview. Inter-rater agreement was very good to excellent for milder levels of suicidal feelings, but problematic for suicide attempts. The latter figure was however based on very few observations, as this is a rare event in population-based studies.

Due to the tendency for unhealthy persons to decline participation in follow-up examinations (Lissner *et al*., 2003), healthy participants may be over-represented in the longitudinal Gothenburg H70 Birth Cohort Study. Three-year mortality was higher in non-participants compared with participants (Karlsson *et al*., 2009). Therefore, it should be noted that the observed prevalence rates of suicidal feelings may be an underestimation of the true prevalence. In addition, persons with suicidal feelings might be more likely to decline further participation or to die and thus not contribute follow-up data. Study participants were examined at different time intervals. Whilst some examinations were performed on a yearly basis, others had several years between follow-up interviews. Also, the number of examinations, which the participant took part in, varied between persons. A further limitation is the inclusion of multiple observations from some participants which means that some of the data are non-independent, which affects the models. As this was a population-based study, it lacked power to specifically study severe suicidal ideation or behaviour. The same was the case for analysis on the potential sex differences in centenarians. Care should be taken in the generalisability of the results to other populations, or to other outcomes such as suicide risk. As demonstrated in a recent meta-analysis (Hubers *et al*., 2018), risk for completed suicide in persons with suicidal ideation varies substantially among different populations. Concerning the assessment of suicidal feelings, a recall bias in distinguishing between past week, past month and past year suicidal feelings cannot be excluded (Olsson *et al*., 2016), also given the fact that participants with mild cognitive impairment were not excluded from analyses. Furthermore, depression diagnosis was based on past month symptoms, a period of time not corresponding to the study of past year and lifetime suicidal feelings. Moreover, reporting suicidal feelings is a sensitive topic where personal aspects and an open atmosphere during the interview matter. As examinations were performed over a 28-year period (1986–2014) where study personnel changed over time, reporting may have been influenced by individual characteristics of the interviewer, the interviewee–interviewer relationship or changing societal attitudes.

This prospective examination of late-life suicidal feelings in observations including a large sample of centenarians demonstrated an increasing prevalence of suicidal feelings with rising age as well as elevated prevalence of suicidal feelings in participants with concurrent depression. Suicidal feelings were observed to occur also in the absence of depression. As consistent affirmative reporting of suicidal feelings over time was rare, our results suggest that suicidal feelings in late- to extreme late-life are not a widespread, normative phenomenon. These results may engage an open dialogue within society on late-life suicide (Van Orden and Deming, 2017), and thereby help to inform the development of suicide prevention programmes.

## Availability of data of materials

Data are available upon request.
